# PLCε regulates podocyte differentiation and TGF-β1 responses via alteration of SMAD2/SMAD3 ratio

**DOI:** 10.1186/s12964-026-02867-3

**Published:** 2026-05-14

**Authors:** Carl J May, Sarah E Hunter, Agnieszka Bierzynska, Lan Ni, Katherine R Bull, Aneesha Bhandari, Gavin I Welsh, Moin A Saleem

**Affiliations:** 1 Bristol Renal, University of Bristol, Dorothy Hodgkin Building, Whitson Street, Bristol, BS1 3NY England; 2 Nuffield Dept of Medicine, Centre for Cellular and Molecular Physiology, Henry Wellcome Building for Molecular Physiology, University of Oxford, Roosevelt Drive, Oxford, OX3 7XB England

**Keywords:** TGF-β1 signaling, PLCε, Podocyte injury, Proteinuria, Glomerular disease, Cell communication

## Abstract

**Background:**

Amongst genetic causes of nephrotic syndrome (NS), mutations in the *PLCΕ1* gene have been described in patients with early onset NS and diffuse mesangial sclerosis (DMS). However, little is known about how these mutations alter podocyte biology although a role in podocyte differentiation has been proposed (Yu S, et al. Exp Mol Med. 52(4):594–603,2020).

**Methods:**

To explore the role of PLCε in podocytes, A conditionally immortalised human podocyte cell line was generated from a patient with early onset NS, due to a nonsense *PLCΕ1* mutation, resulting histologically in diffuse mesangial sclerosis (DMS).

**Results:**

In comparison to wild type podocytes, the *PLCΕ1* mutant podocyte cell has reduced epithelial features, altered actin cytoskeleton and significantly lower levels of the epithelial marker ZO-1.

**Conclusions:**

We demonstrate that PLCε deficiency is associated with functionally impaired TGF-β1 responses with an altered SMAD2/SMAD3 ratio and absent SMAD2 phosphorylation, resulting in loss of the motility response to TGF-β1. We further show that *PLCE1* knockdown in wild-type podocytes recapitulates this phenotype by knock-down of *PLCΕ1* in wild-type podocytes.

This work reveals that disease-causing mutations in *PLCΕ1* result in podocyte dedifferentiation and uncovers a novel association between *PLCΕ1* deficiency and SMAD2/3 signalling signalling in maintaining podocyte differentiation.

**Supplementary Information:**

The online version contains supplementary material available at 10.1186/s12964-026-02867-3.

## Background

The podocyte sits on the urinary aspect of the glomerulus and is thought to be the target cell in the pathophysiology of Nephrotic Syndrome (NS).

Mutations in over 70 genes which explain the pathogenesis of inherited (monogenic) nephrotic syndrome have been identified [1]. Most of these fall into clearly defined categories for example encoding structural components of the podocyte slit diaphragm (nephrin and podocin etc.) or encoding proteins that regulate the podocyte actin cytoskeleton [1].

*PLCΕ1* is one such ‘nephrotic’ gene. PLCε is involved in phospholipid metabolism and hydrolyses phosphatidylinositol 4,5-bisphosphate (PIP2) to generate the downstream signalling messengers diacylglycerol (DAG) and inositol trisphosphate (IP3). Mutations in *PLCE1* are known to cause early-onset nephrotic syndrome and kidney failure in humans [2]. yet global knockout of *PLCΕ1* in a mouse has no renal phenotype, although stressing the mice with hypertension reveals glomerular damage and proteinuria [3]. Mutations in *PLCΕ1* result in diffuse mesangial sclerosis (DMS), a histological variant of NS. in which the mesangium expands leading to damage of the capillary lumen [4].

The mechanisms by which loss of PLCε leads to glomerular disease are not well understood. PLCε has been shown to affect podocyte migration via Rho GTPases and may also influence podocyte differentiation [2]. However, how and to what extent differentiation is altered remains unclear.As a Rho effector PLCε also contributes to cellular motility by regulating actin cytoskeleton dynamics and cell adhesion [5]. *PLCΕ1* is widely expressed and has been cited as being important in podocyte development [6], however no direct interactions with other proteins associated with monogenic NS have been reported, although PLCε has been shown to interact with LIMS1, which in turn is known to bind to Nck2, a podocyte slit diaphragm protein critical for maintaining the filtration barrier [7].

Podocyte dedifferentiation is associated with remodelling of the cortical actin cytoskeleton and acquisition of mesenchymal features, processes that are closely linked to epithelial–mesenchymal transition (EMT). TGF-β1 is a key regulator of EMT and cytoskeletal dynamics in podocytes. Binding of TGF-β1 to the type II TGF-β receptor leads to recruitment and phosphorylation of the receptor-regulated SMADs, SMAD2 and SMAD3 [8]. Phosphorylated R-SMADs associate with the common mediator SMAD, SMAD4 [9], and the resulting complex translocates to the nucleus where it regulates transcription of target genes involved in cell fate and phenotype. Dysregulation of this pathway has been implicated in podocyte injury and disease progression.

The receptor-regulated SMADs, SMAD2 and SMAD3, have distinct and non-redundant roles in TGF-β1 signalling, and their functional outputs can vary between cell types, including fibroblasts and epithelial cells [10]. In these systems, SMAD2 knockdown enhances SMAD3-dependent growth inhibitory responses to TGF-β1 through increased SMAD3 phosphorylation, whereas SMAD3 knockdown promotes SMAD2-dependent migratory responses owing to increased SMAD2 phosphorylation [11, 12]. Although these observations have not yet been directly examined in podocytes, they highlight a functional antagonism between SMAD2 and SMAD3. These findings suggest that the endogenous balance between SMAD2 and SMAD3 may play a key role in determining the qualitative cellular response to TGF-β1 signalling.

In this study we characterised a conditionally immortalized podocyte cell line developed from a patient with diffuse mesangial sclerosis (DMS) with severe, early onset NS, with a homozygous *PLCΕ1* mutation at nucleotide 321 that leads to a stop codon and a severely truncated protein. We observed a mesenchymal cellular phenotype compared to wild-type podocytes. Since SMAD-mediated TGF-β1 pathways are implicated in podocyte fibrosis and epithelial to mesenchymal transformation [13], we explored the hypothesis that TGF-β1 signalling responses are disturbed by this mutation. Here we report alterations in SMAD2 phosphorylation that are associated with altered functional responses of the cells to TGF-β1.

## Materials and methods

### Cell culture

To investigate the impact of a Phospholipase C epsilon 1 (*PLCΕ1*) mutation on podocyte function, signalling and behaviour, a patient derived *PLCΕ1* mutant conditionally immortalised human podocyte cell line was derived, by transfection of primary podocytes from a nephrectomy specimen, with a temperature sensitive SV40 transgene and the hTERT construct (Ethics approval MREC/00/6/02) as described previously [13].

The child had early onset (at age 13 months) steroid resistant nephrotic syndrome, with rapid progression to established renal failure. Parents were known to be consanguineous. The kidney was removed at age 18 months for attenuation of proteinuria.

In the patient derived cell line there are two Single Nucleotide Variants (SNVs) adjacent to each other at nucleotides 320 and 321 (Supplementary Fig. 1). The first SNP leads to a synonymous substitution as both GAG and GAA code for glutamic acid. The second SNP 321 C > T changes the codon from CGA (arginine) to TGA (stop). This premature stop codon leads to the truncation of the mutant PLCε. Both SNPs are homozygous.

Wild-type and *PLCΕ1* mutant podocytes were cultured consistent with established methods as previously detailed [13].

### Western blotting

Cell lysates were extracted from podocytes at day 0 (podocytes that had only been cultured at the permissive temperature of 33 °C) and at day 14 (podocytes that had completed the 14-day differentiation period at the non-permissive temperature of 37 °C). The protein was then extracted using a Triton-X based extraction buffer supplemented with phosphatase and protease inhibitors as previously described. The lysates were run on 10% acrylamide gels. All blots shown are representative of at least three different experiments.

Western blotting was performed using the Bio-Rad Mini-Protean II System and 10% SDS-PAGE gels under reducing conditions. The gels were electrophoretically transferred onto PVDF membranes. Following transfer, the blots were blocked using a 5% Bovine Serum Albumin (BSA) solution. Blots were then probed with primary antibodies: PLCε (HPA015597 Sigma), pSMAD2 (#3101 Cell Signaling Technology), pSMAD3 (#9513 Cell Signaling Technology) ( SMAD2/3 (#5678 Cell Signaling Technology), Beta-Actin (#A4700 Sigma), Nephrin (#ABT331 Merck), Synaptopodin (#ab50859 Abcam), CD2AP (#2135 Cell Signaling Technology), Claudin-1 (#4933 Cell Signalling Technology), ZO-1 (#8193 Cell Signaling Technology), Beta-Catenin (#8480 Cell Signaling Technology) or SMAD2/3(3102 S Cell Signaling Technology). All primaries were used following a 1:1000 dilution in 3% BSA and incubated with the blot at 4 °C overnight.

Primary antibodies were probed using a HRP conjugated species-specific secondary antibody: either rabbit (#A0545 Sigma) or Mouse (#A9044 Sigma). Secondaries were used following a 1:10,000 dilution in 3% BSA and incubated with the blot for one hour at room temperature.

The membranes were incubated with a synthetic analogue of peroxide and with a signal enhancer, which are mixed in a 1:1 ratio (Geneflow K1-0070).

Blots were imaged using the UVP GelDoc-ItTS Imaging System using VisionWorks LS Analysis Software (Ultra-Violet Products LTD, Cambridge, UK).

### Densitometry

The densitometry in this study has been calculated using ImageJ. ImageJ has a dedicated gel analyser that plots an intensity curve within each lane on the gel. The area under the curve is measured. This gives the intensity of the band above the level of background on the blot. For each blot, the intensity of the protein of interest was normalised to the intensity of a housekeeping protein (Beta Actin was used throughout) values were then used for quantitative comparison between conditions.

### Immunofluorescence

Cells were grown on uncoated coverslips and cultured for 14 days at the non-permissive temperature of 37 °C. After this period, the cells were fixed using 4% PFA and permeabilised using a 0.2% triton solution. The coverslips were then blocked in a 5% BSA solution for one hour at room temperature. Primary antibodies were diluted 1:125 in 3% BSA and applied to the coverslips before being incubated overnight at 4 °C. Species specific secondary antibodies conjugated to a FITC or TRITC label were diluted 1:200 in 3% BSA and applied to the coverslips before being incubated at room temperature for one hour. Coverslips were then mounted using VECTASHIELD HardSet Mounting Medium with DAPI (Vector Laboratories). SMAD2/3(3102 S Cell Signaling Technology) and Phalloidin (8953 Cell Signaling) were used to stain podocytes grown on glass coverslips.

### Fluorescent microscopy

Fluorescent microscopy was performed on a Leica CTR 7000 using a Leica DFC7000T camera.

### Nuclear accumulation of R-SMADs analysis

Nuclear translocation of R-SMADs was quantified using ImageJ. Regions of interest were defined based on DAPI staining to delineate individual nuclei. Mean fluorescence intensity in the green channel within each nuclear region was measured as a readout of nuclear R-SMAD signal. To allow comparison between conditions, nuclear green channel intensity values were normalised to the mean nuclear intensity measured in unstimulated cells from the same experiment. This normalisation therefore reflects relative changes in nuclear R-SMAD signal following TGF-β1 treatment rather than absolute basal levels of nuclear R-SMAD. Baseline nuclear signal in unstimulated cells is expected to be minimal and likely reflects background fluorescence or very low basal signalling.

### *PLCΕ1* CRISPR/Cas9 gene editing

3 sgRNA oligos were designed to target *PLCΕ1* exon 1 A: B: TCTACATCATCACAATTATC and C: AGACGCTTTTAAAAGCAAAA, GAATCTCCTGACTAACAAAGand cloned into the LentiGuide-Puro plasmid (lentiGuide-Puro was a gift from Feng Zhang (Addgene plasmid # 52963 ; http://n2t.net/addgene:52963 ; RRID: Addgene_52963) [14]. Lentivirus was generated in HEK293T cells and infected into undifferentiated proliferating immortalised human podocytes which had previously been infected with a viral Cas9 contruct (lentiCas9 Blast a gift from Feng Zhang (Addgene plasmid # 52962 ; http://n2t.net/addgene:52962 ; RRID: Addgene_52962) and stably expressed Cas9. Infected cells were selected with Puromycin and, single clones were isolated using a Sony Sorter SH800S and successful editing confirmed by PCR (primers L AGCAGCTGCAGTGTGATCAT and R CTCTTCTGATCTATGTAACTCCATGC) and TIDE analysis (Brinkman, Chen, Amendola, & van Steensel, 2014). Clones from both B and C sgRNA with frameshift deletions were identified. The G12 line was generated using sgRNA C and has a 2 bp insertion and a 1 bp deletion with < 3% normal allele [14, 15].

### Statistics

Graphpad Prism 9.0 was used to graph and analyse all data. Asterisks are used throughout to denote the following levels of statistical significance: *P = <* 0.05 *, *P = <* 0.01 ** and *P = <* 0.001 ***. Details of specific tests used and p values are detailed in the figure legend.

n refers to independent experiments performed on the same podocyte clone. Statistical analyses were performed in GraphPad Prism. Unless stated otherwise, n refers to independent biological replicates from separate experiments. For comparisons between two matched conditions a paired t test was used; for two independent groups an unpaired t test was used; and for comparisons involving two factors a two-way ANOVA was used with multiple comparisons correction as indicated in the figure legends. Given the small sample sizes in some analyses, we have interpreted non-significant findings cautiously. Additionally the limited number of biological replicates in some analyses, the absence of statistical significance in certain comparisons may reflect limited power rather than true equivalence.

## Results

### *PLCΕ1* Mutant Podocyte Cell Line

**Fig. 1:**
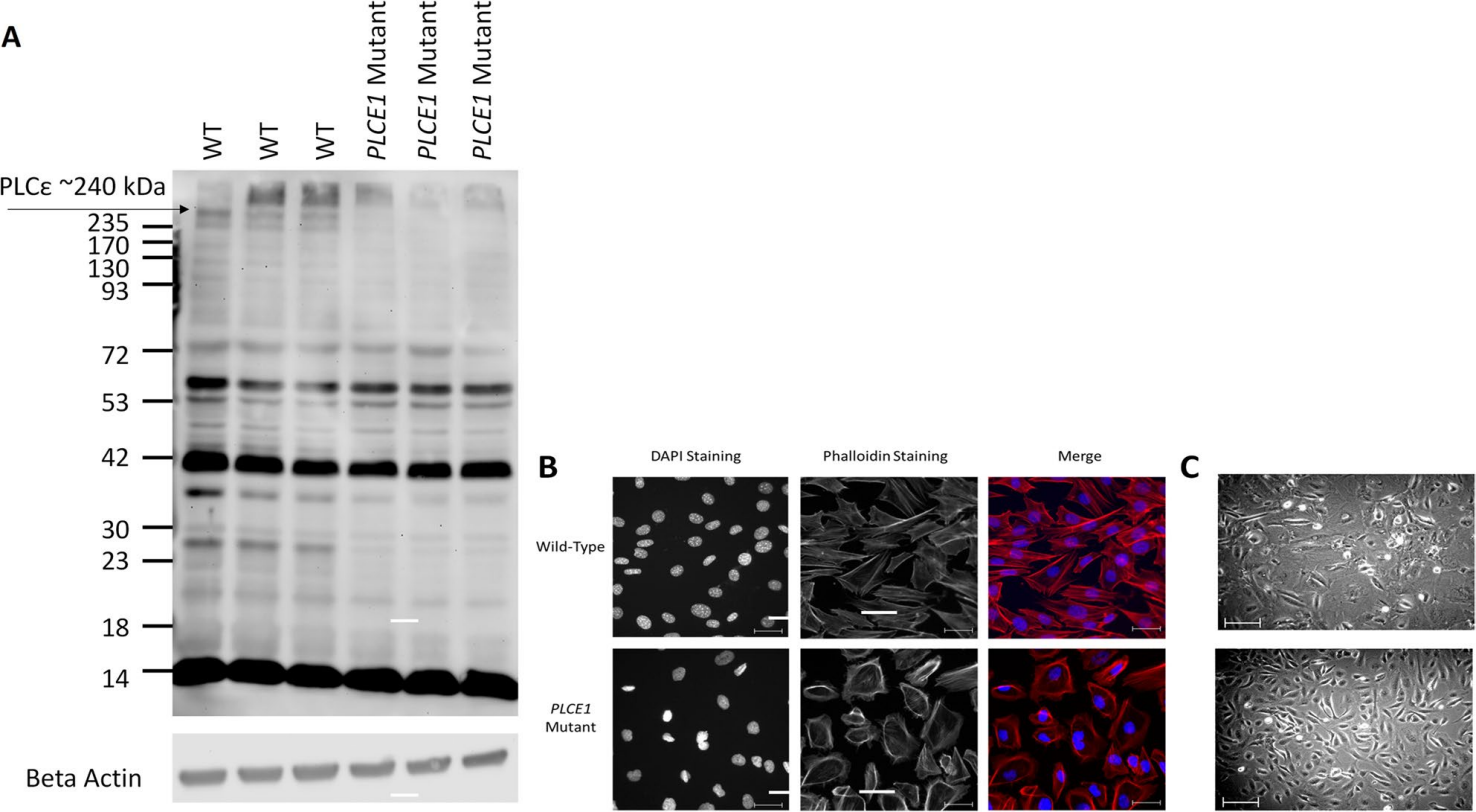
*PLCΕ1* Expression and Morphology of *PLCΕ1* Mutant Podocytes. PLCε Mutant Protein Expression and Cellular Morphology. The human SNV detected in PLCΕ1 leads to the insertion of a stop codon. **A** shows a Western blot to detect PLCε protein in the two cell lines. The antibody used recognises an epitope located downstream of the premature stop codon, and a band at the expected molecular weight for full-length PLCε (~ 240 kDa) is detected in wild-type but not PLCE1 mutant podocytes. Multiple lower molecular weight bands likely reflect non-specific binding by the polyclonal antibody Phalloidin staining shown in Fig. 1**B** shows the different morphology present in the PLCE1 mutant podocytes. Scale bar represents 50 μm. Figure **C** shows a light micrograph of the wild-type and PLCΕ1 mutant podocytes after the 14-day differentiation period The phase-contrast images shown are representative of the respective cell populations. The wild-type podocytes demonstrate the classic arborisation seen in differentiated podocytes that is not present in the PLCΕ1 mutant podocytes. The PLCΕ1 mutant podocytes retain the cobblestone morphology more commonly associated with undifferentiated podocytes. Scale bar 400 μm

The 321 C > T mutation present in the patient-derived podocyte cell line induces a premature stop codon. Using a polyclonal antibody raised against an epitope located downstream of the premature stop codon, a band at the expected molecular weight for full-length PLCε (~ 240 kDa) was detected in wild-type podocytes but not in *PLCE1* mutant cells (Fig. 1A The arrow indicates the band corresponding to full-length PLCε.). A very faint background signal can be observed in the mutant lanes; however, given the presence of an early truncating mutation that eliminates the antibody epitope, this signal is most consistent with non-specific binding rather than detection of PLCε.It is not known whether a truncated form of the protein is expressed or if the mRNA is degraded via Nonsense-Mediated Decay (NMD) Though even if the truncated form persists it will not contain any of the functional domains present in the full length PLCε.

The actin cytoskeleton is central to the podocytes’ ability to form processes and is involved in podocyte motility. Differentiated *PLCΕ1* mutant podocytes demonstrate a morphology that more closely resembles the cobblestone phenotype of the podocytes at 33 °C, the actin cytoskeleton is much more cortical and less arborised (Fig. 1B). We observed several different clones of the immortalised mutant cell lines, with the same morphology, to ensure this was not a clonal effect (data not shown).

### Podocyte marker and epithelial marker expression

**Fig. 2:**
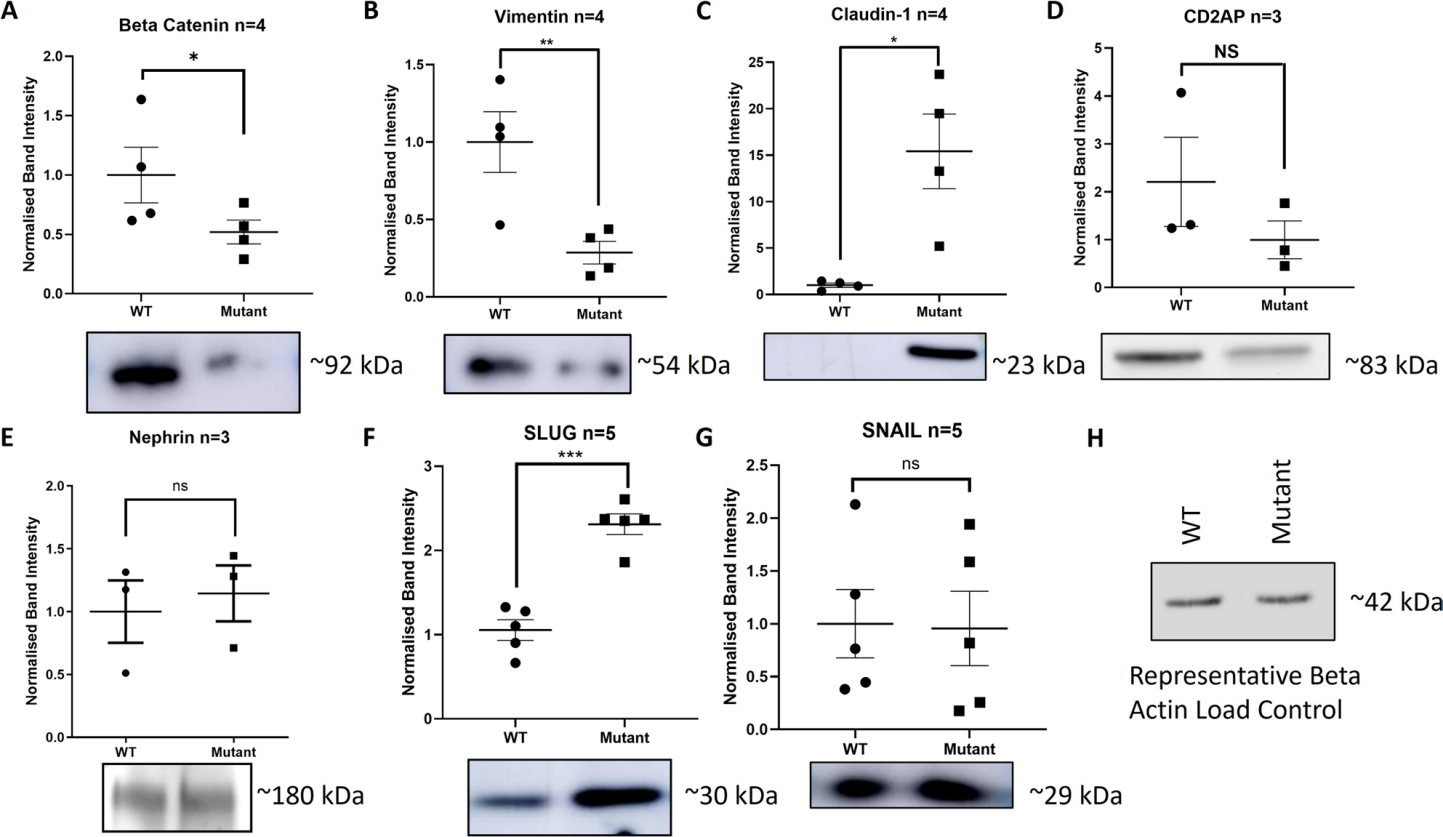
Podocyte Marker and Epithelial Marker Phenotyping of Wild-type and *PLCΕ1* Mutant Podocytes. Densitometry for marker proteins were calculated from at least 3 replicate western blots. The densitometry for each target is shown in the graph with a representative blot underneath. Beta Catenin was significantly reduced in *PLCΕ1* mutant podocytes relative to wild type (**A**) (One-sided paired t-test *P =* 0.0237). Vimentin was significantly reduced (**B**) (One-sided paired t-test *P =* 0.0085). Claudin-1 expression was significantly increased. Claudin-1 expression was significantly increased in *PLCE1* mutant podocytes relative to wild type (C)(One-sided paired t-test* P* = 0.0162) **C** (One-sided paired t-test *P =* 0.0162. There was no significant difference in CD2AP expression between the two cell lines (**D**). There was no significant difference in Nephrin expression between the two cell lines (**E**). Slug is significantly increased in *PLCΕ1* mutant podocytes (**F**) (One-sided paired t-test *P =* 0.0002). Lastly, there was no significant difference in SNAIL expression between the two cell lines (**G**). Densitometric analysis was normalised to β-actin (**H**); representative loading control images are not shown for all blots

The podocyte is a highly specialised epithelial cell. Mature podocytes express the epithelial markers beta-catenin and the intermediate filament vimentin [16]. Expression of both markers was significantly reduced in the *PLCΕ1* mutant podocyte cell line (Fig. 2A and B respectively) (One-sided paired t-Test *P =* 0.0237 and *P =* 0.0085). Claudin-1 is a tight junction protein expressed by dedifferentiated podocytes following loss of the slit diaphragm complex and is not typically present in differentiated podocytes [17]. In keeping with the dedifferentiated phenotype of *PLCE1* mutant podocytes, claudin-1 expression was significantly increased (paired t test, *p* = 0.0162; Fig. 2C).Although podocyte markers are frequently expressed at a reduced level in disease [18], there was no significant reduction in CD2AP expression (Fig. 2D), or in nephrin expression (Fig. 2E). SLUG and SNAIL are transcription factors involved in phenotypic change and maintenance and are most commonly associated with Epithelial-Mesenchymal-Transition (EMT). SLUG expression was significantly upregulated in the *PLCΕ1* mutant podocytes (Fig. 2F One-sided paired t-Test *P =* 0.0002), while there was no significant change in SNAIL expression (Fig. 2G).

### TGF-β1 responses

The cortical actin cytoskeleton of the *PLCΕ1* mutant podocytes suggest that they are more mesenchymal than their wild-type counterparts. Therefore, it was hypothesised that these cells would be hypersensitive to TGF-β1 and undergoing EMT during cell culture.

**Fig. 3:**
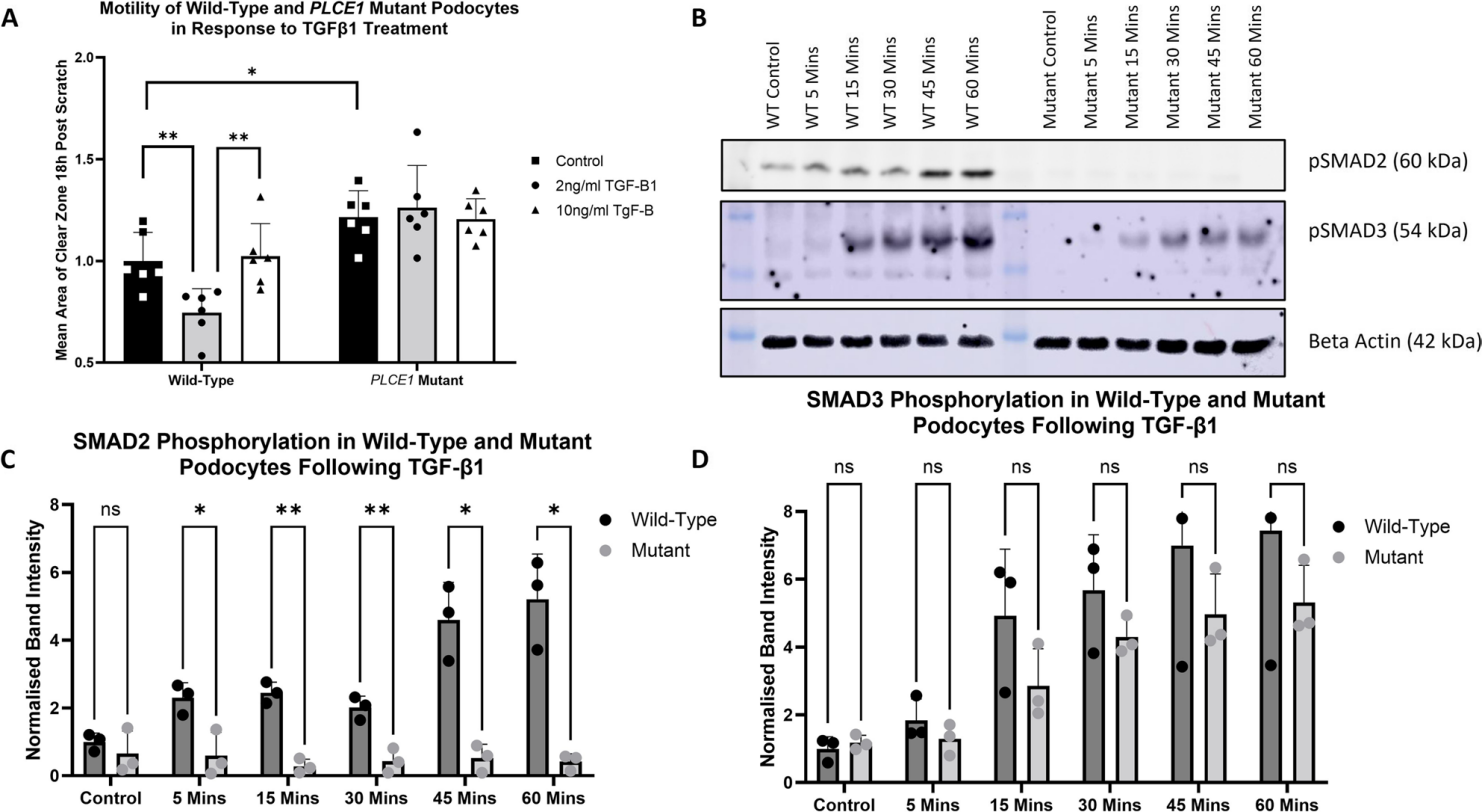
TGF-β1 Responses. **A**. Motility of the Wild-type Podocytes Vs the *PLCΕ1* Mutant Podocytes in Response to TGF-β1 Treatment. Data represent six independent biological experiments, each performed with three technical replicates per condition. Each data point corresponds to the mean value from a single independent experiment, with error bars showing SEM across experiments. Wound closure is expressed as the area of the clear zone at 12 h normalised to the area at 0 h (baseline). Podocytes were treated with 2 ng/ml or 10 ng/ml TGF-β1 as indicated. Figure 3**B** shows representative blots for the densitometry shown in Fig. 3**C** and** D**. Wild-type and PLCE1 mutant podocytes were treated with TGF-β1 (2 ng/ml) for the indicated time points. Panels show densitometric quantification of pSMAD2 (**C**) and pSMAD3 (**D**) from *n* = 3 independent immunoblots, normalised to β-actin. Statistical analysis was performed using two-way ANOVA with genotype and time as independent factors. For pSMAD2, a significant effect of genotype was observed across time points. For pSMAD3, phosphorylation increased over time in both genotypes with no significant genotype effect

Wild-type podocytes became significantly more motile following TGF-β1 treatment at a dose of 2ng/ml, although there was no increase in motility in the wild-type podocytes following TGF-β1 treatment with a dose of 10ng/ml. The mutant podocytes displayed no increase in motility in response to TGF-β1 treatment at either dose (Fig. 3A). Representative images for Fig. 3A are available in Supplementary Fig. 2.

Each of the R-SMADs has specific and non-redundant functions in the TGF-β1 signalling response. Moreover, these roles differ in fibroblasts and epithelial cells [10]. SMAD2 knockdown increases the SMAD3 dependent growth inhibition response to TGF-β1 due to enhanced SMAD3 phosphorylation. Conversely, SMAD3 knockdown increases the SMAD2 cell migration response to TGF-β1 owing to the increased SMAD2 phosphorylation [11, 12]. Although these observations are yet to be replicated in podocytes which is vital due to the known inter cell-type variability in TGF-β1 responses. The enhancement of SMAD3 dependent pathways in the absence of SMAD2 and vice versa demonstrates a functional antagonism between these two R-SMADs. The endogenous ratio of SMAD2 to SMAD3 may be controlling the cellular response to TGF-β1 signalling.

Representative blots of SMAD phosphorylation following treatment with 2ng/ml are shown in Fig. 3B The SMADs mediate the signal of TGF-β1. SMAD2 phosphorylation following TGF-β1 treatment is completely suppressed in *PLCΕ1* mutant podocytes (Fig. 3C two-way ANOVA Cell Line Variation *P =* 0.028). However, both WT and *PLCΕ1* mutant podocyte cell lines phosphorylate SMAD3 following TGF-Β1 treatment, with no significant differences in the signalling between the two (Fig. 3D).

### SMAD2 and SMAD3 nuclear accumulation

Phosphorylated R-SMAD (SMAD2 or SMAD3) translocates to the nucleus, increasing the likelihood of target genes being transcribed. Wild-type podocytes demonstrate the same discrimination between doses of TGF-β1 in terms of their nuclear accumulation of R-SMAD as they did in their motile response, The Wild-type podocytes show significant nuclear accumulation of R-SMAD in response to 2ng/ml but not 10ng/ml of TGF-β1, while the *PLCΕ1* mutant podocytes demonstrated a significant increase in R-SMAD nuclear accumulation in response to both doses of TGF-β1. As total SMAD2 and SMAD3 protein levels were not independently quantified, we cannot exclude the possibility that differences in nuclear SMAD signal partially reflect changes in total SMAD abundance in addition to altered phosphorylation dynamics. Nuclear accumulation of phosphorylated R-SMADs increased following TGF-β1 treatment in both genotypes, with a differential response at higher ligand concentrations in *PLCE1* mutant podocytes.

(4 A-C). Although the antibody used recognised both SMAD2 and SMAD3, only phosphorylated R-SMADs are retained in the nucleus. Therefore, in *PLCE1* mutant podocytes, the nuclear signal is likely to arise predominantly from SMAD3, as these cells show only weak and non-significant SMAD2 phosphorylation. Only a dose of 2ng/ml TGF-β1 significantly increased R-SMAD nuclear accumulation (Tukey’s Multiple Comparison Test *P =* 0.0039). The effects of TGF-β1 on podocyte behaviour were dose dependent. In wild-type podocytes, low-dose TGF-β1 promoted migration, whereas higher doses did not, in line with the observations regarding the nuclear accumulation of R-SMADs. Nuclear translocation indicates pathway activation but does not necessarily predict functional outcome, particularly given the distinct roles of SMAD2 and SMAD3 [2]. The altered SMAD2/SMAD3 balance in *PLCE1* mutant podocytes is therefore likely to shape the qualitative nature of the TGF-β1 response rather than simply its magnitude [1].

### SMAD2/SMAD3 ratio

**Fig. 4:**
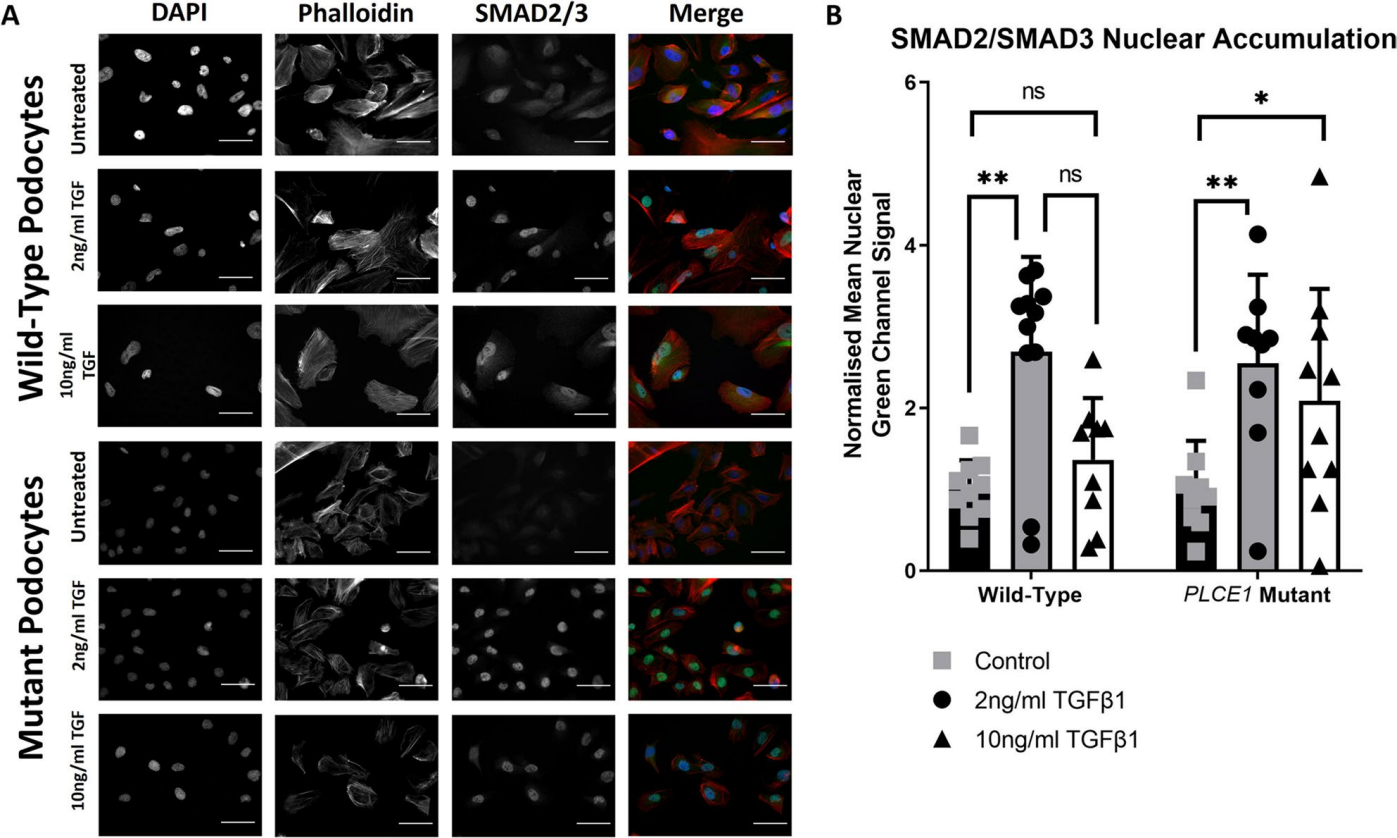
Nuclear accumulation of phosphorylated R-SMADs in response to TGF-β1. **A** Representative immunofluorescence images of wild-type and PLCE1 mutant podocytes stained with an antibody recognising total SMAD2/3 (green) and DAPI to mark nuclei (blue). **B** Quantification of nuclear SMAD2/3 signal. Nuclear regions of interest were defined based on DAPI staining, and mean green channel intensity within each nucleus was measured and normalised as described in the Methods. Data represent mean ± SEM from independent experiments. Statistical analysis was performed using one-way ANOVA with Bonferroni post-hoc testing. Significance is indicated as *p < 0.05, **p < 0.01, ***p < 0.001

There is 3.5 times as much SMAD2 relative to SMAD3 in the wild-type podocytes. However, in the *PLCΕ1* mutant podocytes there is a ~ 1.5:1 ratio between SMAD2 and SMAD3 (Fig. 4).

### Recapitulation of *PLCΕ1 *mutant podocyte cell line phenotype

**Fig. 5:**
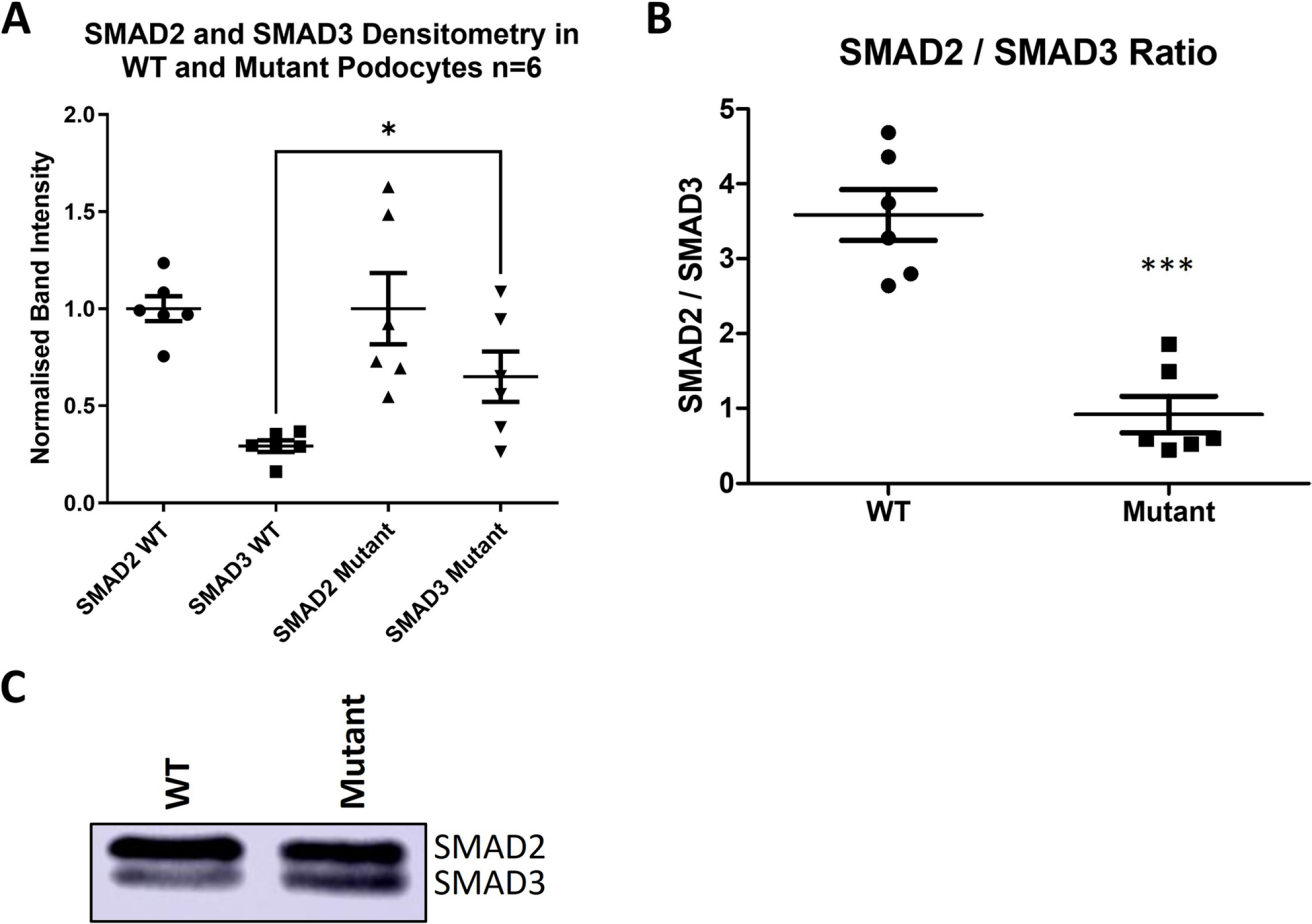
SMAD2/3 Ratio in WT and Mutant Podocytes. SMAD2/3 Ratio in Wild-type Podocytes Vs the PLCΕ1 Mutant Podocytes. Figure 5**A** shows the densitometry for SMAD2 and SMAD3 in WT and Mutant podocytes. The blots are representative of six blots with an example blot shown in 5**C**. The higher band is SMAD2 (52kDA) and the lower band is SMAD3 (48kDa). The Wild-type podocytes have roughly 3.5 times the amount of SMAD2 relative to SMAD3 whereas the PLCΕ1 mutant podocytes have roughly 1.5 times the amount of SMAD2 and SMAD3 (5**B**). There is a highly significant difference between the SMAD2/3 ratios in the wild-type and the PLCΕ1 mutant podocyte cell lines (*P* = 0.0002 One-sided paired T-test). SMAD3 expression is shown relative to SMAD2 from the same samples, such that the data represent a SMAD3:SMAD2 ratio and are internally normalised

Since the *PLCΕ1* mutant cell line was generated from a patient, it is possible that other genetic differences between this and the WT cell line could influence the phenotype. To eliminate this background heterogeneity, *PLCΕ1* was knocked down in the WT cell line using CRISPR/Cas9 technology. A knock down of 90% was achieved as assessed by qPCR (Fig. 5A). the SMAD2/SMAD3 ratio was significantly altered in *PLCE1* mutant podocytes compared with wild-type cells (Fig. 5C; Mann–Whitney test, *p* = 0.002).

**Fig. 6:**
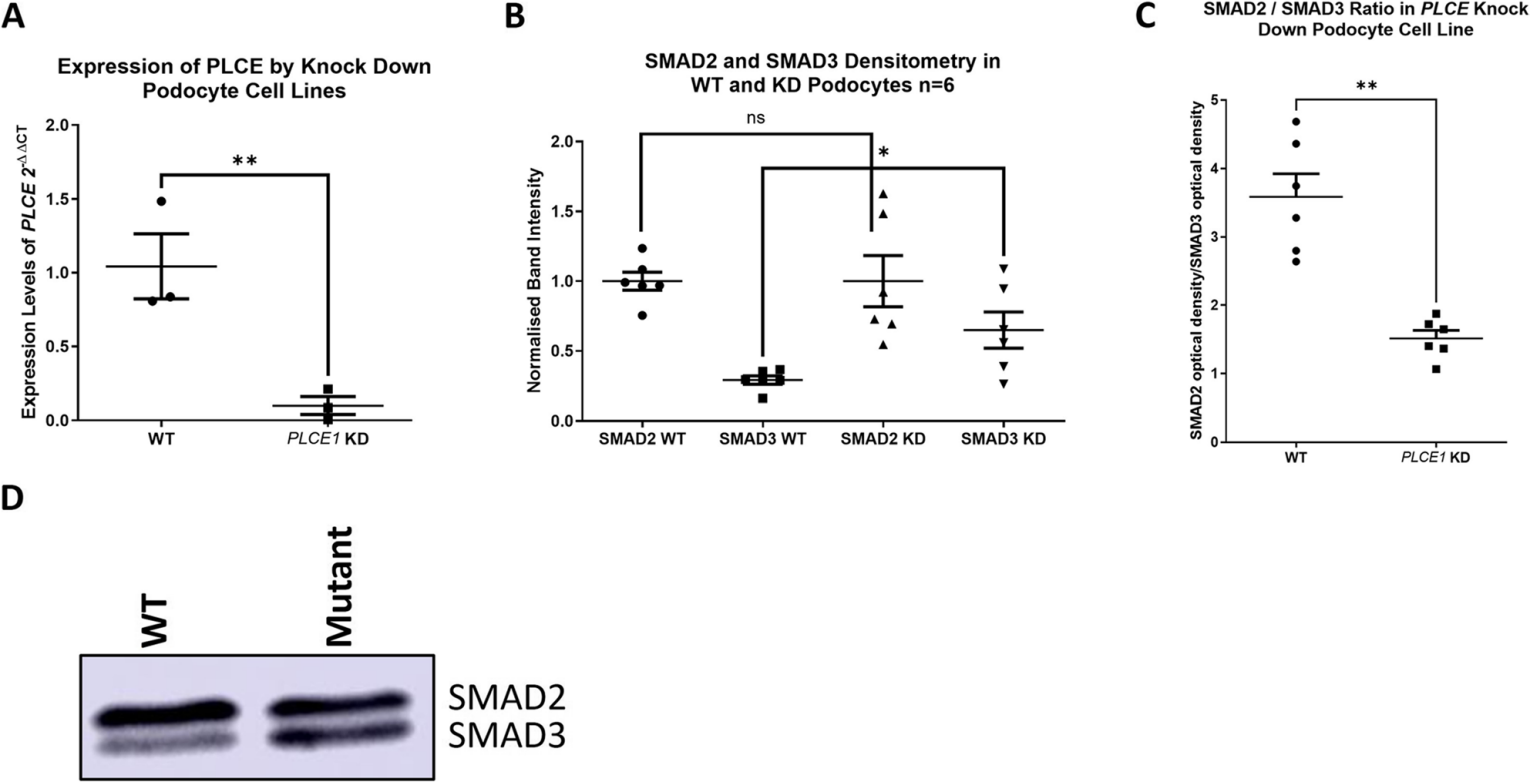
SMAD2/3 Ratio in PLCΕ1 Knock Down Cell Line. PLCΕ1 was knocked down using CRISPR/Cas9 technology. A knock down of 90% was achieved as assessed by qPCR using GAPDH as housekeeper (**A**). This cell line had a SMAD2/3 ratio of ~1:1.5 close to the ratio observed in the PLCΕ1 mutant podocyte cell line (**B** and **C**). A representative SMAD2/3 blot is shown in **D**. SMAD3 expression is shown relative to SMAD2 from the same samples, such that the data represent a SMAD3:SMAD2 ratio and are internally normalised

**Fig. 7:**
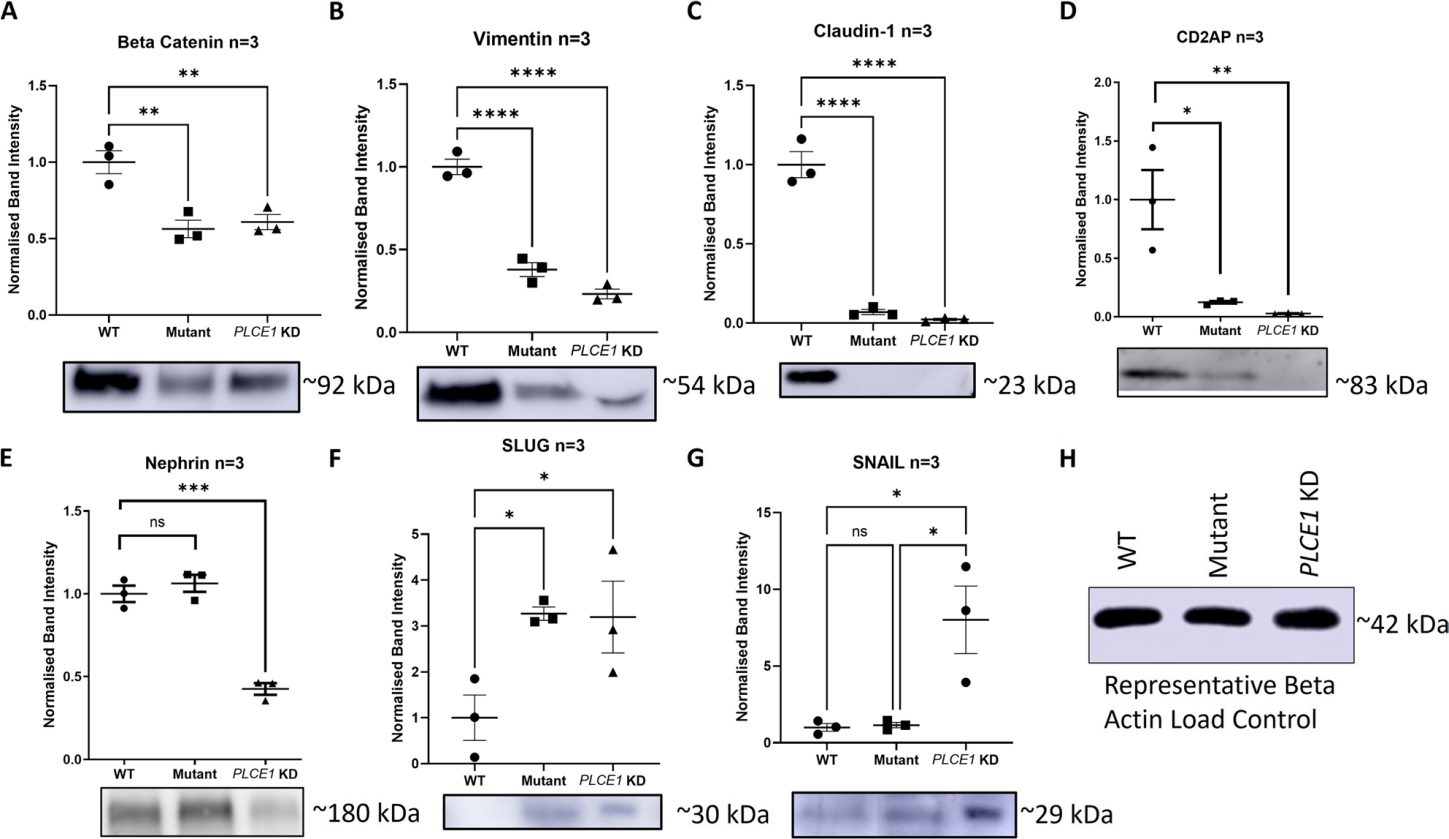
Podocyte Marker and Epithelial Marker Phenotyping of PLCΕ1 Knock Down Cell LineProtein expression of β-catenin (**A**), vimentin (**B**), claudin-1 (**C**), CD2AP (**D**), nephrin (**E**), SLUG (**F**), and SNAIL (**G**) was assessed by immunoblotting and quantified by densitometry using β-actin as a loading control. Densitometric analysis was normalised to β-actin (**H**); representative loading control images are not shown for all blots. PLCΕ1 mutant podocytes showed the same pattern of significant and non-significant changes as observed in Figure 2. Compared with wild-type podocytes, β-catenin (A; P = 0.0043 and P = 0.0072), vimentin (B; *P* < 0.0001 for both), claudin-1 (C; *P* < 0.0001), and CD2AP (D; *P* = 0.0164 and *P* = 0.0100) were reduced in both PLCΕ1 mutant and knockdown cells. Nephrin expression did not differ between wild-type and PLCΕ1 mutant podocytes but was reduced in PLCΕ1 knockdown cells (E; P < 0.0001). SLUG expression was increased in both PLCΕ1 mutant and knockdown podocytes (F; *P* = 0.0438 and *P* = 0.0497), while SNAIL was increased only in PLCΕ1 knockdown cells (G; *P* = 0.0248). Statistical analysis was performed using one-way ANOVA with Bonferroni’s multiple comparison test

In line with observations in the patient cell line (Fig. 2), EMT and differentiation marker expression in the *PLCΕ1* KD podocyte cell line was significantly different when compared to wild-type podocytes and comparable to the *PLCΕ1* mutant cells, with the exception of nephrin and SNAIL which were more altered in the KD cell line compared to WT or mutant cells (Fig. 6). The *PLCE1* KD cell line no longer phosphorylates SMAD2 following treatment with 2ng/ml TGF-β1 (Fig. 7C). This observation mirrors the response observed in the *PLCE1* mutant podocyte cell line.

## Discussion

This study presents the effects of a human *PLCΕ1* mutation in the mature podocyte in vitro. Indeed, we corroborate the findings of Yu et al. that *PLCΕ1* mutant podocytes are less motile than their wild-type counterparts (Fig. 3A) [2]. However, for the first time, we show that an altered SMAD2/3 ratio could have implications for a TGF-β1 mediated cellular differentiation profile in podocytes.

Work in the field so far has focused on the role of *PLCΕ1* in podocyte migration and development [6, 19]. Nephron development is known to proceed along several well characterised stages. These are known as the comma shaped body, the ‘S’ shaped body, the capillary loop stage and finally onto the mature glomeruli [20]. *PLCΕ1* expression is first detected during the ‘S’ shaped stage and is upregulated during the capillary loop stage, where it is required for normal progression. Without functional PLCε, development is halted at this stage, resulting in the histopathological presentation of Diffuse Mesangial Sclerosis (DMS) [21], the same characteristic histology seen in patients with *WT1* mutations, a gene crucially required for nephron development [13].

**Fig. 8:**
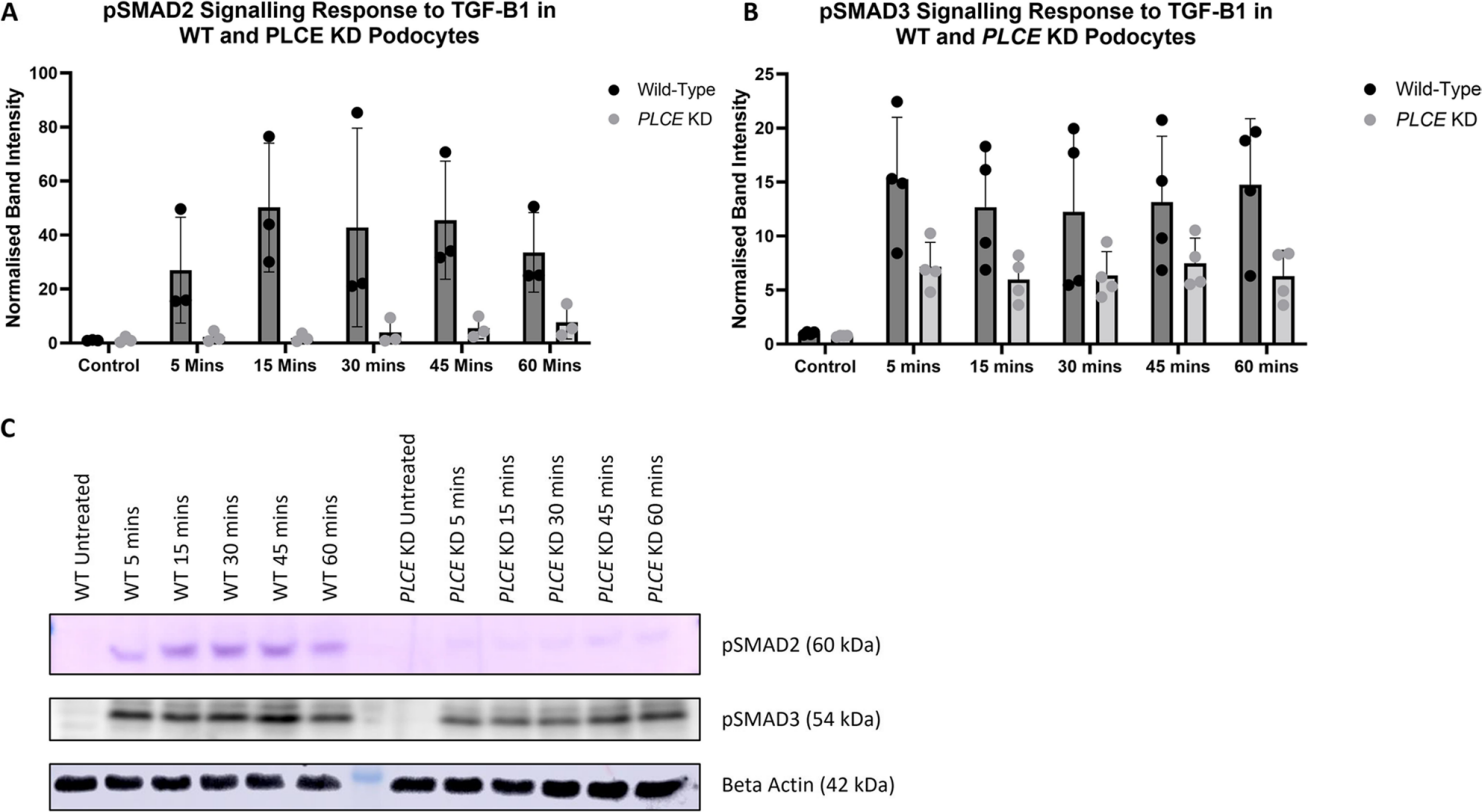
PLCE1 Knock Down in Wild-Type Podocytes Stops the Phosphorylation of SMAD2 in Response to TGF-β1 Treatment. The densitometry shown in **A** demonstrates the a PLCE1 knockdown podocyte cell line no longer phosphorylates SMAD2 in response to 2ng/ml TGF-β1 treatment. Although variability at individual time points limited pairwise comparisons, two-way ANOVA demonstrated a significant main effect of genotype on the pSMAD2 response over time The PLCE1 knockdown podocytes phosphorylate SMAD3 in response to 2ng/ml just as the PLCE1 mutant podocytes do. A representative set of blots for the densitometry shown in **A** and **B** is shown in **C**

This *PLCΕ1* mutant cell line, derived from a patient with diffuse mesangial sclerosis, is less epithelial than wild-type podocytes. The actin cytoskeleton is much more cortical in the *PLCΕ1* mutant podocytes than in the wild-type (Fig. 1B [13]) *PLCΕ1* mutant podocytes demonstrate significant differences in marker expression suggesting that they have a dedifferentiated podocyte phenotype (Fig. 2). We have shown for the first time that *PLCE1* mutation is associated with alterations in the TGF-β1/SMAD signalling pathway, linking PLCε deficiency to changes in this key regulator of cell growth, differentiation, and migration (Figs. 3 and 8). The *PLCE1* knockout mouse model displays impaired keratinocyte motility and poor wound healing [22], suggesting PLCε alters cell motility in vivo. The reduced SMAD2 phosphorylation and subsequent increase in nuclear translocation at high levels of TGF-β1 may be related to the altered ratio between SMAD2 and SMAD3, the two-receptor mediated SMADs that are phosphorylated by the activated TGF-β1 receptor (Fig. 4). The altered SMAD2/SMAD3 ratio in the *PLCΕ1* mutant podocyte cell line was recapitulated by knock-down of *PLCΕ1* in a wild-type podocyte cell line, supporting a consistent association between PLCε deficiency and altered PLCε SMAD2 and SMAD3 expression (Fig. 5). The changes in marker expression seen in the *PLCE1* mutant podocytes (Fig. 2) were largely replicated in the *PLCE1* knockdown podocytes and the amount of SMAD2 relative to SMAD3 was reduced from 3.5 to 1.5 (Fig. 6C) with equal amounts of SMAD2 and SMAD3 having been observed in the *PLCE1* mutant podocytes (Fig. 4B).

Increasing evidence suggests that the receptor mediated SMADs, SMAD2 and SMAD3 have interrelated but distinct functions with SMAD3 demonstrating a profibrotic role [23–27]. Podocyte motility is a SMAD2 mediated response [11], so the lower levels of SMAD2 relative to SMAD3 in the *PLCΕ1* mutant cell line may explain why these cells were significantly less motile at baseline than the wild-type and lacked the increased motility response expected with TGF-β1 treatment (Fig. 3A). Decreased podocyte motility is a known feature of dedifferentiated podocytes, consistent with this the patient derived and edited *PLCΕ1* mutant cells expressed lower levels of key podocyte differentiation markers and increased levels of the EMT transcription factor SLUG (Fig. 2) [28]. It is not clear why the *PLCΕ1* KD cell line demonstrates significantly less nephrin and SNAIL.

Additionally, it seems that the SMAD2 mediated response to TGF-β1 is dose dependent. Wild-type podocytes became significantly more motile following treatment with 2ng/ml, but not 10ng/ml, of TGF-Β1. Differential dose responses have been seen in a mouse model of diabetic nephropathy [29]. Although these doses are not necessarily physiologically relevant, it does show that podocytes can discriminate between doses of TGF-β1 and elicit different responses. *PLCΕ1* mutant podocytes exhibit a SMAD3 mediated TGF-β1 response, and there is no such dose discrimination at the two doses tested.

Overall, this work suggests that PLCε deficiency is associated with alterations in the SMAD2/3 ratio and with impaired podocyte motility and differentiation. This could be explained by differential roles for the two-receptor mediated SMADs in podocytes, under both resting and TGF-β1 stimulated conditions.

PLCε has been shown to interact with Rho GTPase dependent signalling pathways and adaptor proteins linked to the actin cytoskeleton, including NCK2 associated networks. It is therefore possible that the altered TGF-β1/SMAD responses observed in PLCε deficient podocytes arise indirectly as a consequence of disruption to these pathways rather than through direct regulation of SMAD signalling by PLCε itself. While the consistent findings observed in both a patient derived *PLCE1* mutant podocyte cell line and an CRISPR/Cas9 mediated *PLCE1* knockdown podocyte cell line support a relationship between PLCε deficiency and an altered SMAD2/SMAD3 balance, rescue experiments will be required to establish causality and to determine whether PLCε influences SMAD signalling directly or via intermediate signalling pathways.

It remains to be shown how the *PLCΕ1* truncation mutation and *PLCE1* knockdown leads to the abnormal SMAD2/SMAD3 ratio, affecting the TGF-β1 response. PLCε can activate PKC. This is achieved via the generation of the secondary messengers Inositol Triphosphate (IP_3_) and Diacylglycerol (DAG) from Phosphatidylinositol 4,5-bisphosphate (PIP_2_). Via binding to specific calcium channels, IP_3_ can increase intracellular calcium concentrations and activate PKC [26].

There are two phosphorylation sites in the MH1 domain of SMAD3 that are the target of activated PKC [30]. Phosphorylation at these sites inhibits the DNA binding capability of SMAD3. In this way activated PKC can inhibit the SMAD3 mediated TGF-β1 signalling response [31]. We hypothesise that the absence of functional PLCε reduces PKC activity and will result in less SMAD3 inhibition. This may contribute to the observed dominance of the SMAD3-mediated TGF-β1 response in the *PLCΕ1* mutant podocytes. Through the lack of functional PLCε, one potential regulatory mechanism of SMAD3-mediated signalling may be impaired. Although this pathway goes some way to explaining why the mutant podocytes may signal via SMAD3 rather than SMAD2 in response to TGF-β1, it does not explain the difference in SMAD2/SMAD3 ratio between the two cell lines. This proposed mechanism remains speculative and does not exclude the involvement of Rho GTPase– or NCK2-dependent pathways downstream of PLCε.

The SMAD2/3 ratio may be a useful therapeutic target. Switching the bias in favour of SMAD2 is likely to be protective and could alleviate podocyte dedifferentiation. Blockade of the TGF-β1 pathway as a whole would likely cause many side effects due to its pleiotropic effects on cell behaviour: blockade of SMAD3 signalling may well be more clinically useful. Indeed, inhibition of SMAD3 using a specific inhibitor, SIS3, has been shown to block renal fibrosis in a model of diabetic nephropathy [32]. Future work should focus on the clinical utility of SMAD3 inhibition in *PLCΕ1* mutant FSGS, and potentially other forms of glomerular fibrosis.

## Conclusion

We have shown here that a *PLCE1* mutant podocyte cell line has a dedifferentiated phenotype and altered SMAD2/3 ratio. This phenotype could be recapitulated in wild-type podocytes by CRISPR/Cas9 knock down of *PLCE1*. This suggests that *PLCε* deficiency is associated with alterations in the SMAD2/SMAD3 ratio and render the podocyte less epithelial. Drugs targeting the SMAD2/3 axis may represent a strategy to promote restoration of podocyte differentiation in DMS. This should be a priority for future work. 

## Supplementary Information

Supplementary Material 1: Supplementary Figure 1: Sanger Sequencing of PLCΕ1 Mutant Podocyte Cell LineSanger Sequencing detected 2 SNPs leading to the introduction of a premature stop codon. Supplementary Figure 2: Phase-contrast images showing the remaining cell-free area 18 hours after mechanical denudation in wild-type and PLCE1 mutant podocytes treated with vehicle control, 2 ng/ml TGF-β1, or 10 ng/ml TGF-β1, as indicated. Images are representative of six independent biological experiments and illustrate the extent of wound closure quantified in Figure 3A.

## Data Availability

No datasets were generated or analysed during the current study.

## Declarations

### Availabilty of Data and Materials

Data sharing is not applicable to this article since no datasets were generated or analysed during the current study.

## Abbreviations

ANOVA: Analysis of Variance
CRISPR: Clustered Regularly Interspersed Short Palindromic repeats
DAG: Diacyl Glycerol
DAPI: 4’,6-diamidino-2-phenylindole
DMS: Diffuse Mesangial Sclerosis
EMT: Epithelial Mesenchymal Transition
FITC: Fluorecein Isothiocyanate
HRP: Horse Radish Peroxidase
IP_3_: Inositol Triphosphate
NMD: Nonsense Mediated Decay
NS: Nephrotic Syndrome
PCR: Polymerase Chain Reaction
PFA: Paraformaldehyde
PIP_2_: Phosphatidylinositol 4,5 bisphosphate
PKC: Protein Kinase C
PLCE1: Phospholipase C epsilon gene
PLCε: Phospholipase C epsilon protein
qPCR: Quantitative Polymerase Chain Reaction
R: SMAD-Receptor Mediated SMAD
sgRNA: Single Guide RNA
TGF: β1-Transforming Growth Factor Beta 1
TRITC: Tetra Methylrhodamine
WT: Wild-Type
