# Supplementary material for: PLCε regulates podocyte differentiation and TGF-β1 responses via alteration of SMAD2/SMAD3 ratio
Source: Cell Commun Signal. 2026 May 14;24:391. doi: 10.1186/s12964-026-02867-3 (PMC13340390; doi:10.1186/s12964-026-02867-3)
Supplement: Supplementary file 1 — Supplementary Material 1: Supplementary Figure 1: Sanger Sequencing of PLCΕ1 Mutant Podocyte Cell LineSanger Sequencing detected 2 SNPs leading to the introduction of a premature stop codon. Supplementary Figure 2: Phase-contrast images showing the remaining cell-free area 18 hours after mechanical denudation in wild-type and PLCE1 mutant podocytes treated with vehicle control, 2 ng/ml TGF-β1, or 10 ng/ml TGF-β1, as indicated. Images are representative of six independent biological experiments and illustrate the extent of wound closure quantified in Figure 3A. [file 12964_2026_2867_MOESM1_ESM.docx]

**Supplementary Figure 1:** Sanger Sequencing of PLCΕ1 Mutant Podocyte Cell Line

Sanger Sequencing detected 2 SNPs leading to the introduction of a premature stop codon.


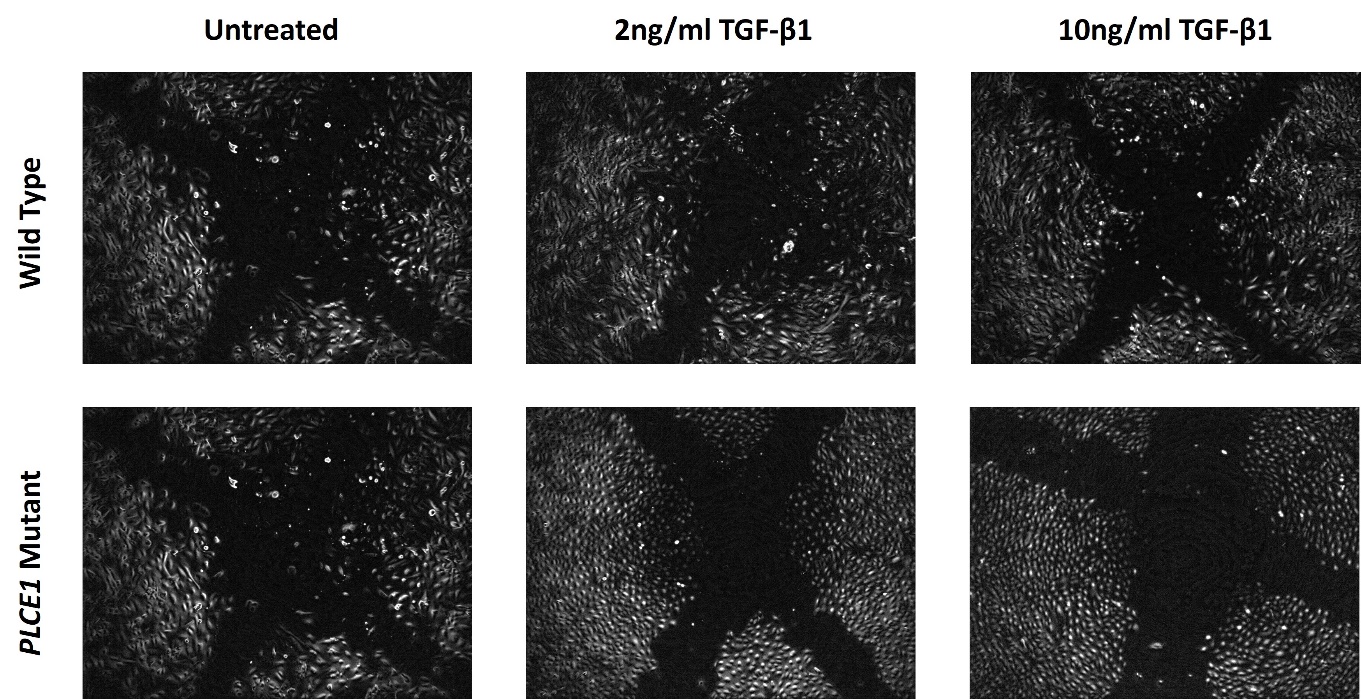


**Supplementary Figure 2**: Phase-contrast images showing the remaining cell-free area 18 hours after mechanical denudation in wild-type and *PLCE1* mutant podocytes treated with vehicle control, 2 ng/ml TGF-β1, or 10 ng/ml TGF-β1, as indicated. Images are representative of six independent biological experiments and illustrate the extent of wound closure quantified in Figure 3A.
